# Implementation of a Learning Management System for Medical Students: A Case Study of Kilimanjaro Christian Medical University College

**DOI:** 10.15694/mep.2017.000050

**Published:** 2017-03-15

**Authors:** Dativa Tibyampansha, Glory Ibrahim, Gibson Kapanda, Chrispina Tarimo, Amani Minja, Ahaz Kulanga, Charles Muiruri, Kien Mteta, Egbert Kessy, John Bartlett

**Affiliations:** 1 Kilimanjaro Christian Medical University College; 2 Duke University

**Keywords:** Learning Management System(s), e-learning, medical education, Blended learning

## Abstract

This article was migrated. The article was marked as recommended.

Learning Management Systems (LMS) are powerful tools for the organization and presentation of curricular learning materials, for monitoring of student and faculty members performance, and for overall quality control. However, there is limited evidence regarding the acceptance and performance of LMS in Africa. This manuscript describes the implementation, the outcomes, and the challenges of the first five years of a LMS at the Kilimanjaro Christian Medical University College (KCMUCo). The LMS has been fully adopted into KCMUCo curriculum and deployed to 1356 students. It has been demonstrated to enhance learning with strength of consensus measure of 84% for basic sciences and 78% for clinical classes. 80% of faculty members have been satisfied with the use of LMS. Electronic assessment has become an obligatory platform for theory examinations. LMS adoption may improve education outcomes at other medical schools in Tanzania and sub-Saharan Africa.

## Background

Schools of medicine in Africa face challenges resulting from increasing class sizes, faculty shortages, inadequate infrastructure and limited financial resources. Medical schools are a major component of health care systems, primarily for training and increasing the number medical doctors ( Chen et al., 2012). In addition, they train other health care workers such as dentists and nurses. Despite the extraordinary need for these health care professionals, medical schools in sub-Saharan Africa face challenges in meeting the demand, with limited numbers of medical schools producing insufficient numbers of graduates. According to Mullan et al. (2011), 169 medical schools in 48 countries of sub-Saharan Africa produce some 10,000 graduates per year. However, this number is insufficient to meet the recommended ratio of 1:5,000 ( Makasa, 2009), and it is also adversely affected by brain drain, unequal geographical distribution, and pursuit of non-medical employment. Medical schools also face shortages of faculty, while the existing faculty are typically overwhelmed with a multitude of duties, facility deficits, and drastic increases in the enrollment number of students without a commensurate increase in faculty growth ( Mullan et al., 2011). These factors are likely to compromise the quality of health education and hence the competences of the trained health care professionals.

Tanzania has only 6 medical schools, with the majority of which are privately owned. In 2013, the doctor-to-population ratio in Tanzania was 1:20,000 as reported by the World Health Organization (WHO), that is far below the recommended ratio of 1:5,000 ( Makasa, 2009). Recognizing the importance of producing quality health care workers, in 2010 the United States President’s Emergency Fund for AIDS Relief (PEPFAR) through Medical Education Partnership Initiative (MEPI) offered a five-year, $10 million grant to Kilimanjaro Christian Medical University College (KCMUCo) in partnership with Duke University and the Duke Global Health Institute. One of the MEPI specific objectives was to “enhance the quality and quantity of medical education” (“About MEPI,” n.d.). To achieve this objective, KCMUCo embarked on increasing the number of enrolled students, introducing a learning management system (LMS), and transforming the culture of teaching and learning by heavily investing in upgrading the information communication technology (ICT) infrastructure.

Learning Management Systems (LMS) are software applications that are used to manage online learning and teaching such as delivery of electronic curricular learning materials and tracking, reporting of learning processes ( Asiri et al., 2012; De Smet et al., 2016) LMS were invented in the late nineties and have gained widespread adoption since then. The latest 2016 4th Annual LMS Data Update by the “4th Annual LMS Data Update | edutechnica,” (2016) confirms that more than 90% higher institutions in United States, Australia, Canada, and United Kingdom actively use LMS

This paper describes the process of implementation of a LMS at KCMUCo, including resource requirements, institutional support, faculty and student engagement and lessons learnt after five years of implementation.

## Steps to successful implementation

### Theoretical framework development

We utilized the New Zealand e-Learning Planning Framework (eLPF) to evaluate our e-learning capability (Te Toi Tupu Consortium, 2014). eLPF provides a roadmap of processes and practices that have been validated as critical in building an institutional e-learning capability. It describes four phases from emerging, engaging, extending to empowering on how technology can be adopted and integrated into teaching and learning. At the emerging phase, an institution focuses on investigating, raising awareness and planning while at the engaging phase, an institution focuses on plan establishment. The extending phase is reached when processes and practices are effectively aligned and at the empowering stage technologies make new ways of learning possible. The eLPF identifies the core components to be addressed at each phase, and these include institutional support, faculty and student engagement, technical expertise, and infrastructure and support systems. The engagement of each of these components will be explained in the subsequent sections.

### Institutional support

The KCMUCo leadership provided support and sustainability plans to practices and policies established under MEPI. One of them was the development and implementation of an incentive compensation plan to recognize faculty effort and time in using LMS. The impact of incentive plans was assessed based on the number of faculty using LMS over time.

### Infrastructure upgrade

The Information and Communication Technology (ICT) infrastructure at KCMUCo was improved with installation of a centralized campus area network connected to the Internet via the high-speed fibre-optic SEACOM cable. The initially purchased bandwidth of 10 mbps was increased to 30Mbps in 2016 with government assistance and price subsidization. Renovation and expansion of student and faculty computer laboratories was undertaken. All students were offered access to the computer laboratories equipped with 270 computers. Other improvements included 8 audio-visual-enabled lecture theatres, one wet laboratory for experiment demonstrations, and an automatic standby generator to provide power during power cuts. All medical students were provided with mobile computing devices (tablets).

### LMS selection

A number of LMS solutions, both proprietary and open source learning, were assessed. The proprietary software Learning Content Management System (LCMS+) was selected, as it was specifically designed to address the needs of medical education. LCMS+ was designed and built at the Duke University School of Medicine (DUSOM) beginning in 2005, and has been commercially available since 2010. The LCMS+ has been used by our partner (DUSOM) and by several other medical schools in the United States. The selection was followed by the installation of LCMS+ and by training of support specialists.

### Staff hiring

The principal qualities considered upon hiring the LMS experts included training capabilities, customer care skills, teamwork, multi-tasking, ability and readiness to learn new skills, and ICT expertise. These qualities helped in the formation of a strong team of three individuals with training backgrounds including sociology, computer science, public health and health informatics. The LMS experts worked closely with a team of five ICT experts at the back-end to ensure that the system runs smoothly. Various educational and LMS trainings were regularly provided, including trainings at the Duke University School of Medicine and in strategic plan development. Two specialists received support towards degrees in MSc Health Informatics and MSc Information Technology, respectively.

### Faculty and student enrollment

At the beginning of every academic year, new (i.e Year 1) students were taken through a two- to four-hour orientation on how to use the LMS, including guiding materials such as brochures, flyers, manual, and video tutorials. Six hours per week were set aside for one-on-one consultations with faculty and students. Faculty and staff members additionally had orientations one month before the academic year commenced. They were also offered regular (quarterly) departmental orientations and one-on-one consultations upon demand. To fully involve the older, less computer-savvy faculty, a work plan was instituted with steps and procedures to follow in order to ensure a successful course set-up. Particularly active faculty members served as examples to showcase the advantages of LMS and to encourage their peers.

The initial plan was to have the LMS fully utilized by all medical students and faculty within five years. Between 2011 and 2016 the LCMS+ was introduced to five consecutive freshmen classes, and each class continued its use in subsequent years. Recruitment of faculty started with those involved in teaching the Year 1 class. Expansion proceeded consecutively to other classes as the student cohorts advanced each year until all medical school faculty were covered.

Thus, LCMS+ was introduced throughout the entire 5-year medical curriculum. LCMS+ has also been adopted by non-medical student curricula such as the Bachelor of Science (BSc) in Health Laboratory Sciences, Optometry, Physiotherapy and Nursing.

### Monitoring and evaluation

To monitor progress and impact of the LCMS+, feedback on user perception, usage and acceptance both from faculty and students was regularly obtained utilizing electronic surveys, observation, interviews, and by extraction of user activity statistics from LMS such as login hits and number of active faculty.

To evaluate the individual behavioural perceptions and intentions to use LMS, we used the Technology Acceptance Model (TAM) developed by ( Davis, 1989). In this model, lack of acceptability of an innovation is a major challenge to implementation ( Davis, 1993). The two main characteristics of TAM are perceived usefulness (PU) (the extent to which a person believes that using a technology will enhance her/his productivity), and perceived ease of use (PEU) (the extent to which a person believes that using a technology will be free of effort). These factors directly influence the behavioral intention to use new technology. The behavioral intention to use technology is also influenced indirectly by external variables through PU and PEU. Separate multivariate regression with robust standard errors and Mann-Whitney U test were used to test the following hypotheses;

H1: Age, gender and entry qualifications had no effect on PU and PEU, H2: PEU had no effects on the PU, H3: PEU had no effect on attitude towards using LMS (AU), H4: PU had no positive effect on AU, H5: PU had no effect on behavioural intention to use LMS (IU), H6: AU had no effect on IU, H7: Frequency of use of LMS had no effect on students’ grade point average.

### Back-up plan

Back-up is a critical part of planning for any institution running computerized information systems. Institutions may suffer serious consequences and inconveniences if there are no good and effective on- and off-site back-up systems. One back-up server was set up to back-up all data from the primary server. Since the back-up server was set not to mirror the live character of the primary server, and the examination system is proprietary and developers are off-site, there emerged a need to implement another back-up examination system that would be completely controlled and upheld by the on-site LMS and ICT experts. In 2014 an open source LMS (Moodle) was adopted as a back-up examination system. The back-up assessment system has successfully worked from that point forward to date. This implementation has ensured the integrity, protection and confidentiality of electronic examinations.

## Outcomes

Based on the eLPF , KCMUCO is currently in transition from extending to empowering phase. LMS has been integrated across the entire KCMUCo community. LMS has been incorporated into curriculum and has facilitated other teaching methods such as team-based learning and problem-based learning (TBL, PBL). There are three courses actively using LMS to facilitate TBL with an average of about 180 students per course. Students and faculty have been able to utilize the LMS at anytime and from anywhere if an internet connection is available.

### Content delivery

**Figure 1.**
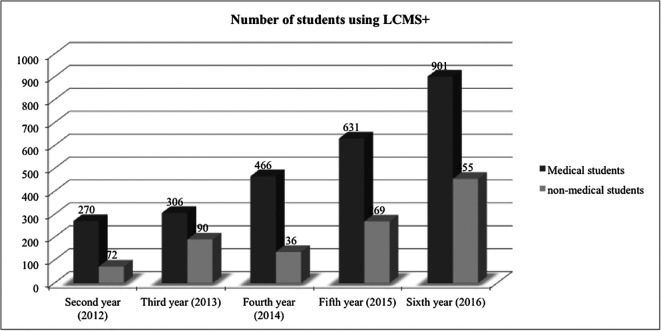
LCMS+ usage among students over five years (2012-2016)

**Figure 2.**
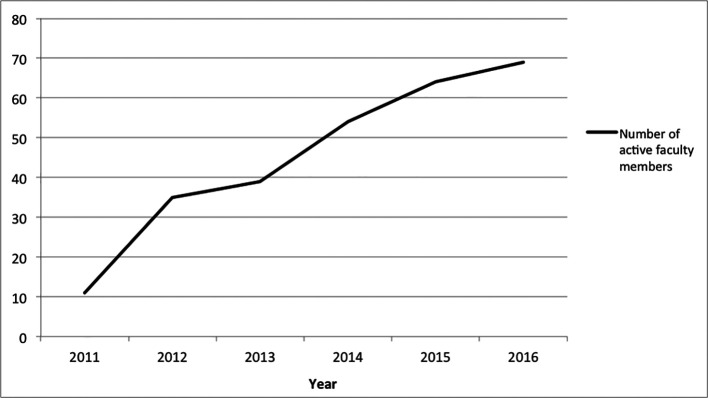
Faculty participation over time

We started implementing LMS in 2011 with 150 first-year medical (MD1) students and five faculty members. Three months after implementation (January 2012), LMS had registered 220 students, including postgraduate students. In late 2016, there were 901 medical, 107 postgraduate, 49 BSc. Physiotherapy, 154 BSc. Nursing, 129 BSc. Health Laboratory Sciences, and 16 BSc. Optometry students, making a total of 1356 out of 1782 (76%) KCMUCO students actively utilizing the LMS ( Figure 1). For faculty, the target was to have at least 70% of all basic science faculty and 40% of clinical faculty using LMS after five years. As a result of the perceived benefits of LMS and faculty incentives, the number of actively basic science faculty after five years was 81% and 46% for clinical faculty, for a total of 69 active faculty members ( Figure 2).

The target set for the five years of implementation was to have all 17 basic sciences (MD1&MD2) and 16 clinical sciences (MD3, MD4 & MD5) courses available and active in the LMS. This goal was achieved with 100% success.

### Electronic assessments

The administration of examinations at KCMUCo had traditionally been paper-based. The adoption of electronic assessments at KCMUCo was undertaken to assist faculty with the burden of student examinations, which had previously been graded by hand.

The LCMS+ examination system was adapted to host electronic assessments. To begin with, an electronic assessment work plan and a standard operating procedure were developed to help guide the stakeholders on the steps to go through to ensure efficient and opportune arrangements of electronic exams. A number of workshops were developed for faculty on the optimal methods of creating meaningful electronic assessment questions, particularly multiple-choice questions. Both internal and external experts facilitated these workshops. The commonly used types of questions were multiple choice, true and false, matching, and free response questions. The initial target was to have all basic and clinical science course (MD1 to MD5) examinations administered after five years of implementation. To date, 88% of basic science courses and 69% of clinical science courses have reached the target.

### Learning enhancement

**Figure 3.**
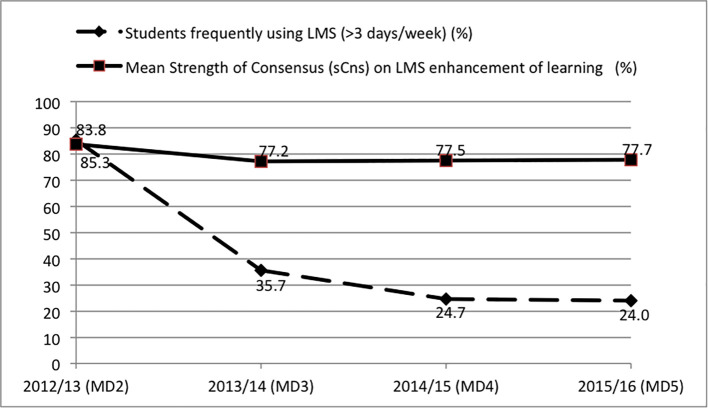
Student frequency of use of LMS and consensus on enhancement of learning

The first cohort of students to be introduced to LMS was monitored for all five medical school years on the use of LMS. The frequency of use of LMS per week and the mean strength of consensus measure on LMS enhancement of learning over the years from second year of study through fifth year are shown in Figure 3. We observed a declining trend in the frequency of use of LMS per week upon the transition from basic science years (year 1 and year 2) to clinical sciences years (year 3 to year 5). This was attributed mainly due to lower utilization of LMS by clinical sciences faculty, and also to more time spent on hands-on practical learning with patients. However, although agreement as measured by the strength of consensus measure (sCns) slightly declined in response to the survey question that LMS enhances medical learning during the first clinical sciences year (year 3) (from 84% in year 2 to 77% in year 3) there was a small (non-significant) increasing trend over the years up to fifth year of study (rising from 77% in year 3 to 78% in the fifth year). These observations suggest that there is general agreement that the use of LMS enhances medical learning.

### Acceptability of LMS

To determine the influence of actual use on performance, we captured the individual logons of LMS and the grade point average (GPA) for all MD1 students after four months. A questionnaire using an electronic tool ( http://www.surveymonkey.com/) was administered to MD1 students in the academic year 2013/2014. The questionnaire which was an adapted version of the basic TAM questionnaire developed by Davis (1993), was administered to students after they had been trained on the use of LCMS+ and they had used the system for approximately three months.

**Figure 4.**
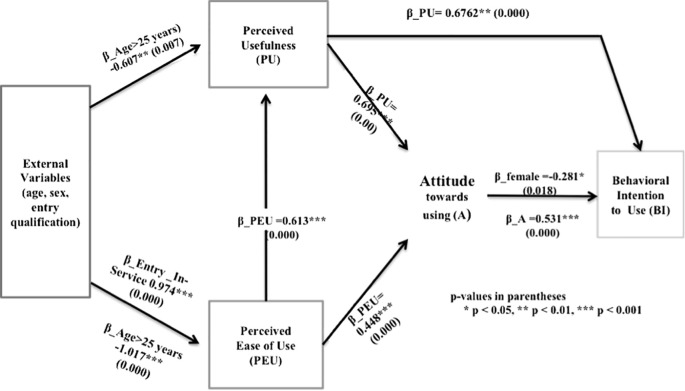
Results of regression analysis of TAM

**Figure 5.**
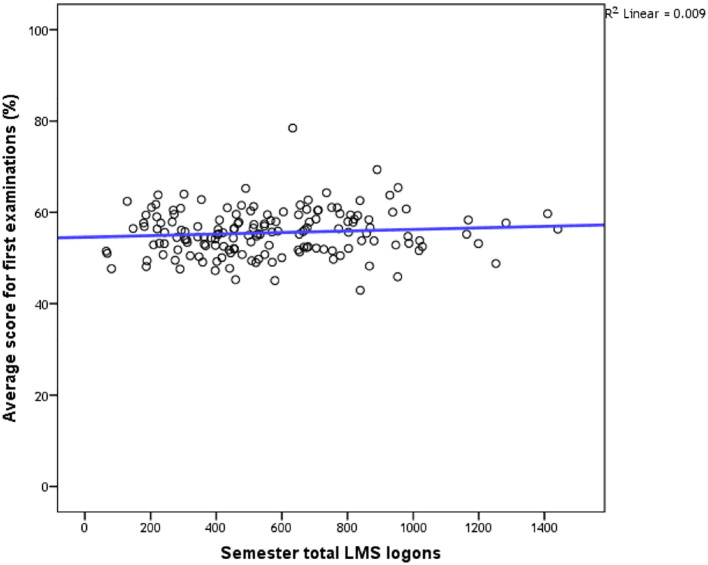
Scatter plot on relationship between LCMS+ logons per student and academic performance (percent average scores)

The general TAM relationships as established by Davis (1989) were mostly consistent with the findings in our study ( Figure 4). Perceived usefulness was a significant predictor of attitudes and subsequent intention to use (p<0.05). However, perceived ease of use independently predicted attitude (p<0.05) but did not predict intention to use. Students who were in-service (not directly from school and have been practicing in medical-related fields before joining medical school) and older than 25 years had lower perception regarding usefulness and ease of use of LCMS+. Gender differences were found in favor of male students with the intention to use construct (p<0.05). Although not significant, higher frequency of use was associated with a slight increase in receiving a higher grade point average in the courses that were using the LMS. Our findings suggest that implementers should pay close attention to older students, in-service and female students. In contrast, although not significant, higher frequency of use was observed with a slight increase in receiving a higher grade point average in the courses that were using the LMS (R ^2^ = 0.009) ( Figure 5). In summary, we concluded that in order for institutions in Sub-Saharan Africa (SSA) to embrace e-learning, it is critical to evaluate technology acceptance since it has long been noted as a challenge in implementation effectiveness and sustainability.

### Faculty satisfaction with LMS

**Figure 6.**
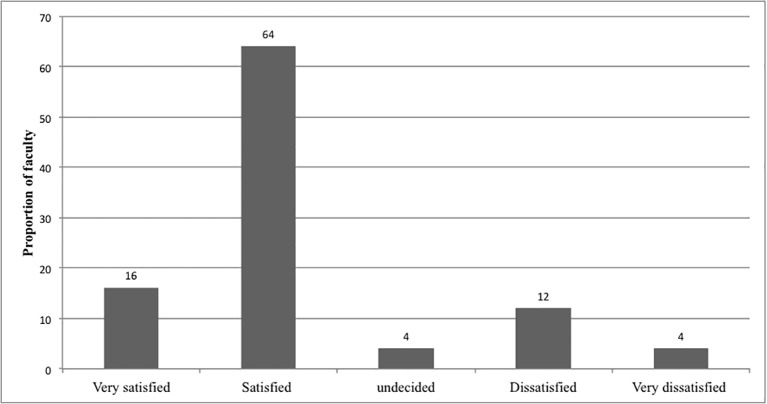
Faculty satisfaction with LCMS+ in medical teaching

Overall satisfaction with the use of LMS at KCMUCo was high; 80.0% of faculty members were either satisfied (64.0%) or very satisfied (16.0%) with LMS as shown in Figure 6. Faculty who were not satisfied with LMS were asked for their opinions on how they can be satisfied, and the majority indicated need for more training sessions on the LCMS+ system and for the involvement of all faculty within KCMUCO (not only faculty teaching medical students).

### Faculty perceived usability of LMS in medical teaching

Faculty who said they had used LMS were further asked to indicate their level of agreement with statements related the usability of LMS in facilitating teaching. The strength of consensus measure (sCns) ranged from 44.8% with respect to the statement “Using LMS alone is sufficient to implement the course I teach” to 93.3% with respect to the statement “LMS is able to enrich my course content through its ability to include resources from Internet”. The mean strength of consensus score was 80.3% with 76% of faculty agreeing that LMS is useful in medical teaching ( Table 1).

## Discussion

We have described the path to successful implementation of a LMS at KCMUCo. KCMUCo is currently in transition from extending to empowering phase of LMS implementation. We feel the use of LMS at KCMUCo is appropriate as users are satisfied with it, and by high student and faculty satisfaction levels. It has taken a strong ICT infrastructure and support systems, with the right mix of human resources, student and faculty engagement, and strong institutional support. Each of these components identified by the Te Toi Tupu Consortium (2014) is very critical in implementation of e-learning at any institution. Lack of cross-functioning collaboration among these components can be a threat to a successful implementation and sustainability of e-learning ( Frehywot et al., 2013; Vovides et al., 2014).

The implementation of LMS at KCMUCo has been very helpful for students, supported by the students’ high-level consensus of learning enhancement, (84% in basic sciences and 78% in clinical sciences). Despite the decline trend from basic sciences to clinical sciences, which is most likely due to more practical sessions during clinical years, the results indicate an agreement on the enhancement of learning. Similar variation in the usage of e-learning between basic science and clinical science have been reported by Ruiz (2006), whereby he found e-learning to be highly variable among medical schools, and it appears to be more common in basic science courses than in clinical clerkships.

Bolliger (2009) has emphasized on the importance of continuous assessment of faculty satisfaction as one the major factors of quality delivery of electronic content. This is important because the success of electronic content delivery relies on the commitment and willingness of faculty to use technology. Faculty at KCMUCo were found to be satisfied with LMS in teaching with sCns=80%, and 76% agreement that LMS is a useful tool in medical teaching. However, they also indicated need for extra training (seminars and tutorials) to make them more conversant and competent, as lack of training related to using technology in teaching can become a major barrier to the integration of technology in an institution ( Asiri et al., 2012b). This kind of training indicated by KCMUCo faculty members has also been emphasized by other researchers ( Vovides et al., 2007) on how to effectively design LMS and best use their learning features.

The increase in the participating faculty over time at KCMUCo ( Figure 2) is clear evidence of the usefulness of the system in the delivery of curricula.

The burden of teaching and grading a growing number of medical students with shortage of faculty while preserving the quality of their training is faced by most academic institutions in resource-constrained settings ( Frehywot et al., 2013). Prior to the introduction of electronic assessments at KCMUCo, many faculty members had been overwhelmed with grading paper examinations, which took them at least one to two months per class. This led to delays in feedback to students, and there was no chance for tracking, verifying and analyzing the assessment. Paper-based examinations were additionally seen to be costly for the College, as they required a great deal of staff support and time to grade. According to Ruiz et al., (2006) a considerable number of publications in the non-medical literature have shown that e-learning can bring about significant cost-savings in relation to reduced instructor training time, travel costs, labor costs, reduced institutional infrastructure, and the possibility of expanding programs, compared with traditional instructor-led learning, sometimes by as much as 50%. Instant feedback generated soon after student submission of the completed examinations has resulted in a substantial reduction of the workload for faculty; the time needed for grading of examinations, and has made reporting easier and quicker at KCMUCo.

For institutions in sub-Saharan Africa (SSA) to embrace LMS, it is critical to evaluate technology acceptance since it has long been noted as a challenge in implementation effectiveness and sustainability. TAM has been proven by substantial number of studies ( Asiri et al., 2012b; Park, 2009; Saadé et al., 2007) to be a useful tool in helping to understand and explain behavioral intention to use e-learning. Our TAM findings ( Muiruri et al., 2014) suggest that implementers should pay close attention to older students (>25 years) and female students as age and gender could negatively affect technology acceptance.

### Success

LCMS+ has increased access to learning content, interactivity, and individualization of choices concerning the form, pace, and blend of learning. Course management from outside KCMUCo is beginning to facilitate teaching certain critically under-staffed elements of the curriculum. The LMS has enhanced and facilitated the communication between students and faculty, and it is used to execute modern and resource-efficient teaching concepts such as team-based learning. The administration has obtained a tool for a fast and objective evaluation of the performance of students, individual departments, and faculty. Taken together, this innovation has rapidly transformed the culture of teaching and learning at KCMCUo.

### Challenges

Limited student and faculty ICT literacy initially created fears of extra workload and resulted in occasional resistance. Non-adherence of faculty to timelines for submitting examinations has been a challenge to the LCMS+ staff. There has been a large increase in the number of students without an equivalent increase in the number of LMS staff. Training opportunities clearly explaining the LMS and its advantages were the most critical components in the implementation o

## Conclusion

LMS at KCMUCo has assisted in handling the burden of increased student enrollment, which has outpaced increases in faculty numbers, and has organized curricular materials in a manner helpful to students and faculty. It has proven to be an important tool in enhancing medical and other health professions education in a resource-limited setting. The applied solutions at KCMUCo are pioneering and the experiences gathered are important to develop similar solutions for other medical schools and disciplines in resource-limited settings.

## Take Home Messages

• Provision of right environment is the key to changing culture of teaching and learning.
• Learning Management Systems are important tools in enhancing medical education and other educational disciplines in resource limited setting despite few challenges faced.

## Notes On Contributors

Dativa Tibyampansha, involved in implementation of the Learning Management System (LMS) and writing up of the manuscript.

Glory Ibrahim, participated implementation of the LMS and writing up of the manuscript. (Dativa and Glory contributed equaly in this manuscript)

Gibson Kapanda, analyzed the data.

Chrispina Tarimo, was involved in monitoring of the implementation of the system and evaluation of the LMS.

Amani Minja, contributed in implementation of the LMS.

Ahaz Kulanga, was involved in conception, planning and implementation of the LMS and critical review of the draft manuscript

Charles Muiruri, was involved in conception and planning and implementation of the LMS and critical review of the draft manuscript

Kien Mteta, participated in planning, conception and excecution of the study.

Egbert Kessy, contributed in conception, planning and execution of the LMS.

John Bartlett, contributed in the conception,planning and implementation of the LMS and also proofreading and critical review the draft manuscript.

## Appendices

**Table 1.** Faculty strength of consensus measuring the usefulness of LCMS+ at KCMUCo in 2014

LCMS+ aspect	Number	Strongly Agree	Agree	Neutral	Disagree	Strongly Disagree	Mean (SDev)	sCns (%)
		n (%)	n (%)	n (%)	n (%)	n (%)		
It is easy to plan a creative course on LCMS+	14	9 (64.3)	4 (28.6)	0 (0.0)	1 (7.1)	0 (0.0)	4.5 (0.9)	90.0
LCMS+ is able to enrich my course content through its ability to include resources from Internet	13	11 (84.6)	1 (7.7)	0 (0.0)	1 (7.7)	0 (0.0)	4.7 (0.9)	93.3
There is very little I can change on the overall visual design of LCMS+	14	0 (0.0)	7 (50.0)	4 (28.6)	2 (14.3)	1 (7.1)	3.2 (1.0)	61.7
The use of hyperlinks provides enrichment to course content	13	5 (38.5)	4 (30.8)	2 (15.4)	2 (15.4)	0 (0.0)	3.9 (1.1)	77.3
Using LCMS+ alone is sufficient to implement the course I teach	14	0 (0.0)	2 (14.3)	3 (21.4)	9 (64.3)	0 (0.0)	2.5 (0.8)	44.8
LCMS+ improves my communication with students	14	10 (71.4)	3 (21.4)	1 (7.1)	0 (0.0)	0 (0.0)	4.6 (0.6)	92.9
The ability to organize the content into categories with LCMS+ makes my teaching more organized	14	9 (64.3)	5 (35.7)	0 (0.0)	0 (0.0)	0 (0.0)	4.6 (0.5)	93.1
The quick and detailed view features of LCMS+ provide a clear layout of the information architecture of the course	14	6 (42.9)	8 (57.1)	0 (0.0)	0 (0.0)	0 (0.0)	4.4 (0.5)	89.0
**Mean**							4.1 (0.5)	80.3
