# Supplementary material for: Implementation of a Learning Management System for Medical Students: A Case Study of Kilimanjaro Christian Medical University College
Source: MedEdPublish (2016). 2017 Mar 15;6:50. [Version 1] doi: 10.15694/mep.2017.000050 (PMC10885243; doi:10.15694/mep.2017.000050)
Supplement: Peer review history

# Reviewer report: Reviewer response for version 1

**DOI:** 10.21956/mep.19458.r28675

**Published:** 2017-03-18

**Authors:** Nandalal Gunaratne

**Affiliations:** 1 Faculty of Medicine, Wayamba University of Sri Lanka

This review has been migrated. The reviewer awarded 4 stars out of 5 This is a well written paper. I also appreciate your rigorous approach to implementation. I am sure this has much to do with your success. I am very interested in the use of Moodle for exams. Is it possible to contact a resource person in your team to find out more? Thank you.

Reviewer Expertise:

NA

No decision status is available

# Reviewer report: Reviewer response for version 1

**DOI:** 10.21956/mep.19458.r28674

**Published:** 2017-03-16

**Authors:** Michael SH Wan

**Affiliations:** 1 University of Notre Dame

This review has been migrated. The reviewer awarded 4 stars out of 5 Thanks for writing up this paper describing the adaption of a new LMS system in the School. As a new medical school, we shared similar experience in the development and implementation of the system as well as the many challenges. The uptake of the LMS by the students depends on the UI and the stability of the system as well as the school Wifi network. While the use of the LMS in assessment was described in detail, would be great to learn about the various learning resources that are available on the current LMS (e.g. lecture capture, group discussion forum for PBL/TBL, blogs, wiki pages,...) and any plan for future additions of new components. The description of the back up server (using Moodle) especially for assessment was very important for us to learn from. Would be great to see a follow up study on the continue improvement in the students learning experiences and course outcomes in the future.

Reviewer Expertise:

NA

No decision status is available

# Reviewer report: Reviewer response for version 1

**DOI:** 10.21956/mep.19458.r28677

**Published:** 2017-03-16

**Authors:** Dujeepa D. Samarasekera

**Affiliations:** 1 National University of Singapore

This review has been migrated. The reviewer awarded 3 stars out of 5 Good paper highlighting the value of leveraging on technology to effectively promote learning and improve satisfaction. Another interesting fact is the use of LMS in a resource scarce training environment. Enjoyed reading the paper.

Reviewer Expertise:

NA

No decision status is available

# Reviewer report: Reviewer response for version 1

**DOI:** 10.21956/mep.19458.r28678

**Published:** 2017-03-16

**Authors:** Ken Masters

**Affiliations:** 1 Sultan Qaboos University

This review has been migrated. The reviewer awarded 3 stars out of 5 A well-written article describing a well thought out and planned implementation. The institutional support and backing is most encouraging, as that is a frequent point of failure. Much of this paper could serve as a blue-print for others wishing to implement LMSs and other learning technologies.I would, however, liked to have seen more detail on how the LMS was used. For example, while I assume there was some distribution of notes, I would like to know if activities like chat rooms, blogging, wiki-creation, interaction with other sites and apps, etc., had been used. Quite a lot of information is given about assessment, and there is mention of using it to support TBL & PBL, but very little detail has been given.Also, I think your discussion could benefit from a more detailed assessment of the tests of your hypotheses.

Reviewer Expertise:

NA

No decision status is available

# Reviewer report: Reviewer response for version 1

**DOI:** 10.21956/mep.19458.r28676

**Published:** 2017-03-15

**Authors:** Richard Hays

**Affiliations:** 1 James Cook University

This review has been migrated. The reviewer awarded 4 stars out of 5 This paper id interesting because it reports the introduction into a low resource setting of a resource that many medical students in the developed world take for granted. The approach to evaluating the impact is sound and the results demonstrate the advantages of supporting learning with a learning management system where there was previously no such system. One weakness is that there is no comparison between different systems, so I am not sure exactly what this LMS feels like from the learners perspective, but that was not possible in these circumstances. While none of the systems available is perfect (and complaints not infrequent), this paper shows that perhaps any system, with student and faculty buy-in and appropriate support, is a valuable and important asset.

Reviewer Expertise:

NA

No decision status is available

# Reviewer report: Reviewer response for version 1

**DOI:** 10.21956/mep.19458.r28679

**Published:** 2017-03-15

**Authors:** Trevor Gibbs

**Affiliations:** 1 AMEE

This review has been migrated. The reviewer awarded 4 stars out of 5 I very much enjoyed reading this paper, which was well written, clear and carefully structured. I feel that it demonstrated well the advantages that a Learning Management System can bring and the overall support it provides. I am sure that other readers in similar situations will learn from reading this paper.I hope that there is a continuation of this study in looking at the long term development of the students and their program, which will I hope provide a micro picture of the LMS advantages

Reviewer Expertise:

NA

No decision status is available
